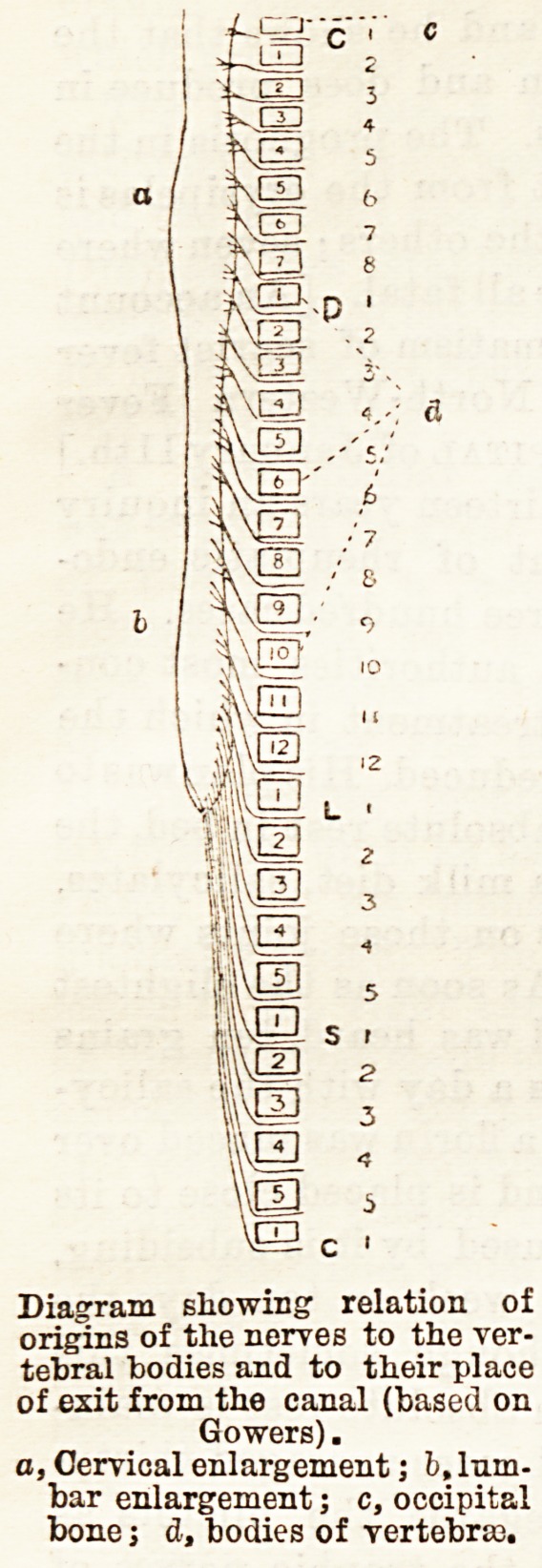# The Localisation of Tumours of the Spinal Cord Amenable to Surgical Treatment

**Published:** 1896-02-08

**Authors:** E. Percy Paton

**Affiliations:** Hon. Physician Mildmay Hospital, Shoreditch.


### MEDICAL PROGRESS AND HOSPITAL CLINICS.

[*The Editor will be glad to receive offers of co-operation and contributions from members of the profession. All letters should be addressed to* THE EDITOR, AT THE OFFICE, 428, STRAND, LONDON, W.C.]

### THE LOCALISATION OF TUMOURS OF THE SPINAL CORD AMENABLE TO SURGICAL TREATMENT.

By E. PERCY PATON, M.D., M.S., F.R.C.S., Hon. Physician Mildmay Hospital, Shoreditch.

Since the diagnosis of the presence of a tumour of the spinal cord, apart altogether from its localisation, is by no means always easy, and since in many cases if the diagnosis of tumour can be arrived at, the localisation is a matter of comparative simplicity, it will be well first to consider those signs and symptoms upon which a diagnosis of new growth rests.

Tumours of the cord may at once be divided into two kinds, viz., extra and intra dural, according to whether they are situated inside or outside the theca, and although this difference may not give rise to any very marked difference in the symptoms, still it is well to bear it in mind. As a rule, in fact in the great majority of cases, the first symptom of tumour is pain, most often situated in the back over the seat of the growth, but not unfrequently radiating along the nerves which arise from this part of the cord. The acuteness and violence of this pain is very typical, and is often such that it cannot be efficiently controlled, even by considerable doses of opiates, and speedily renders the sufferer's life a burden to him. Subsequent to the pain the march of symptoms is usually gradual, but steadily progressive, except in cases where inflammation arises round the growth, which may afterwards subside, and so cause retrogression of symptoms. First comes motor paralysis, giving rise to paraplegia, which often progresses from above downwards, and is followed by sensory palsy, which advances in the opposite direction. A most typical condition, when it occurs, is the presence of hemiparaplegia, with subsequent transference of the symptoms from one side to the other. It is most often seen in intradural cases, and gives rise at first to the symptoms of unilateral section of the cord, which are motor paralysis of the half of the body on the side of the lesion, with hyperæsthesia of the same area, while the opposite half is anæsthetic. There is usually also a narrow band of anæsthesia above the hyperæsthetic area, and of hyperæsthesia above the anæsthetic area. (See diagram.)

As the growth increases, complete loss of sensation and motion results. This train of symptoms is more often seen in intradural than extradural cases, and is never seen when the growth is so low down as to involve the corda equina.

So soon as the cord is so pressed on or involved in the growth that complete paraplegia results, other signs speedily follow which need only be mentioned here; they are exaggerated reflexes, spasms with clonus, rigidity, affection of the bladder, and micturition, &c. These, however, as will be further discussed later, do not all occur if it is the corda equina which is affected. There are also, too, at this stage some physical signs in the column itself, such as localised tenderness over the tumour, stiffness, and some curving of the column, usually lateral. By a careful consideration of the above signs and symptoms, and by giving special weight to the amount and nature of the pain, the transference of symptoms from one side to the other showing the march of the effects of the growth, with at the same time absence of signs of Potts' disease of the spine, the presence of new growth as against myelitis can usually be established; this is also aided by what will now be said with regard to localisation of the lesion, as if it can be shown that only one part of the cord is affected, this goes in favour of the presence of new growth.

The localisation of the seat of the lesion may be easy or difficult. The first step is to determine if the corda equina is affected or not. This can usually easily be determined, as when it is involved, even though the affection should be, as it most usually is, bilateral, causing palsy in both legs, the paralysed parts will not show any signs of rigidity or exaggerated reflexes, ankle or knee clonus, &c., but will instead have flaccid

Diagram showing the anæsthetic, hyperæsthetic, and palsied areas when only half cord pressed on left side affected.

*a*, Anæsthetic areas; *b*, hyperæsthetic areas; *c*, hyperæsthetic area, with motor palsy.

muscles, which rapidly waste, and show the reaction of degeneration to electrical stimulation, the most prominent features of which are diminution or absence of irritability of the muscles to faradic currents, while galvanic stimulation more easily produces an effect. Reaction of the muscles to different kinds of stimuli is altered at the same time, contraction of a muscle being produced with a weaker current by closing the circuit with the anode than with the kathode, thus reversing the normal condition in this respect.

We must now deal with the effects of new growth in different regions of the cord.

In the cervical region pain is usually very marked, and often extends down into the arms; rigidity also of the portion of the vertebral column corresponding to, or just below, the situation of the growth, is usually much more marked here than in other parts of the cord; but here as elsewhere when the cord itself is affected, what gives the clearest indication of the situation of the growth is the upper limit of the anæsthesia, and in this connection a difference is noticed in extradural and intradural cases, for in the latter class of cases the growth is usually situated somewhat higher than one would be led to expect by the upper level of anæsthesia; this was very well shown in one of the earliest cases operated on by Victor Horsley, and is probably due to the upward course of the sensory fibres after they enter the cord, and is found not only in the cervical but also in the dorsal and lumbar regions. A subsidiary aid to localisation is the presence of any rapid wasting (in contradistinction to wasting due to disuse) occurring in the arm as this will indicate that either the nerve root, or the cells in the anterior horns of grey matter corresponding to the nerves which supply the wasted muscles have become involved in the growth. Reflexes, too, corresponding to the region of the cord pressed on and rendered functionless will, of course, be abolished.

In the dorsal region rigidity is usually less marked, but spinal tenderness more so, and this gives some indication of the situation of the growth, while the anæsthetic level gives the same aid as before. Another thing noticeable in this region is the sense of constriction, which is often present, or what are known as girdle pains. Although generally no marked wasting can be observed in any particular muscles, an indication of the condition corresponding to it will be found in the abolition of the trunk reflexes which are related to the portion of cord involved.

The effects produced by interference with the lumbar region of the cord are complicated by the crowding together of the origins of the lumbar and sacral nerves at their connection with the anterior horns of grey matter in the lumbar enlargement, the extent of which corresponds to about the bodies of the tenth, eleventh, and twelfth dorsal and the first lumbar vertebræ.

If the upper part of the enlargement is affected the whole leg as far up as the top of the thigh is paralysed to sensation and motion, including the flexors of the hip and extensors of the knee, some of which muscles may show wasting; there is also loss of knee-jerk due to affection of the reflex centre, while below the knee there is complete palsy, but the reflexes are exaggerated, and ankle clonus is present. When the lower part of the enlargement is involved the flexors of the hip and extensors of the knee may remain intact; while the muscles of the leg and back of the thigh are involved; and, at the same time, the micturition and defæcation centres are interfered with, giving rise to palsy of the bladder and sphincters.

The distinction between affection of the cord itself and the *corda equina* can usually be made by keeping in mind the facts referred to in this connection above. The signs, of course, may be unilateral, but they are usually not so, and are often very variable. The fact that the pain is usually bilateral is a great help in the early stages in separating tumour of the corda from sciatica, as in the latter case the pain is very rarely found to affect both legs. The situation in the corda itself will be best diagnosed by attaching most localising importance to the muscles and sensory and reflex areas in the area which is paralysed, the nerves passing to or from which pass out highest up from the vertebral canal, as this will lead one to the highest point to which the interference with the function of the nerves of the corda has reached. The

Table showing the Relation of the Spinal Nerves to Spinal Muscles and Sensory and Reflex Areas.

| | Motor. | | | | Sensory. | | Reflex. |
|---|---|---|---|---|---|---|---|
| | C1 | | | 1 | | 1 | |
| Sterno-mastoid | 2 | | | 2 | Scalp | 2 | |
| Upper neck muscles | 3 | Lev. ang. scap. | | 3 | | 3 | |
| Upper part of trapezius | 4 | Diaphragm | Shoulder muscles | 4 | Neck and upper part of chest | 4 | |
| | 5 | | | 5 | Shoulder | 5 | |
| | 6 | Serratus<br>Flex. of elbow<br>Supinators | | 6 | Outer side arm | 6 | |
| Lower neck muscles<br>Middle of trapezius | 7 | Extensors of wrist and fingers | | 7 | Radial side of forearm and hand | 7 | Scapular. |
| | 8 | Extensors of elbow<br>Flex. of wrist and fingers<br>Pronators | | 8 | Arm, inner side<br>Ulnar side forearm and hand | 8 | |
| | O1 | Hand muscles | | 1 | Tips of fingers | 1 | |
| | 2 | | | 2 | | 2 | |
| | 3 | | | 3 | | 3 | |
| | 4 | | | 4 | Front of thorax | 4 | |
| | 5 | | | 5 | | 5 | Epigas'ric. |
| Lower part of trapezius and dorsal muscles | 6 | Intercostals | | 6 | Ensiform region | 6 | |
| | 7 | | | 7 | | 7 | |
| | 8 | | | 8 | | 8 | |
| | 9 | | | 9 | | 9 | |
| | 10 | Abdominal muscles | | 10 | Abdomen | 10 | |
| | 11 | | | 11 | | 11 | Abdominal. |
| | 12 | | | 12 | Buttock | 12 | |
| | L1 | | | 1 | Upper part | 1 | |
| | 2 | Cremoster | | 2 | Front of groin and scrotum | 2 | Cremoster. |
| | 3 | Flexors of hip | | 3 | | 3 | |
| Lumbar muscles | 4 | Extensors of knee<br>Abductors of hip | | 4 | Thigh | 4 | Knee. |
| Peroneus and flex. and extensors of ankle | 5 | Extensors and abductors of hip | | 5 | Leg, inner side | 5 | Gluteal. |
| | S1 | Flexors of knee | | 1 | Lower part of buttock | 1 | Foot-clonus |
| | 2 | Intrinsic muscles of foot | | 2 | Back of thigh, leg, and foot | 2 | Plantar. |
| | 3 | Perineal and anal muscles | | 3 | | 3 | |
| | 4 | | | 4 | Perineum and anus | 4 | |
| | 5 | | | 5 | | 5 | |
| | C1 | | | 1 | Skin from covex to anus | 1 | |

Diagram showing relation of origins of the nerves to the vertebral bodies and to their place of exit from the canal (based on Gowers).
a, Cervical enlargement; b, lumbar enlargement; c, occipital bone; d, bodies of vertebræ.

nerves which correspond to any particular group of muscles or sensory or reflex area can be found by reference to the accompanying table, which is extracted from Gowers. The situations at which they leave the vertebral canal will be seen from the accompanying diagram, in which their relation to the bodies of the vertebræ is represented, it being remembered that the tip of the spines of the vertebræ in the lumbar region almost corresponds to middle of the bodies of the corresponding vertebra. This is not the case in the dorsal region, where the tips of the spines, speaking roughly, about correspond to the middle of the body of the vertebra below, while in the cervical region, where the spines can be felt, they correspond again to the bodies, except the last one, or perhaps two. More exact data can be got on the subject of the relation of the spines to the origin of the spinal nerves from Reid, but is, perhaps, hardly necessary for purposes of operation.

## Figures and Tables

**Figure f1:**
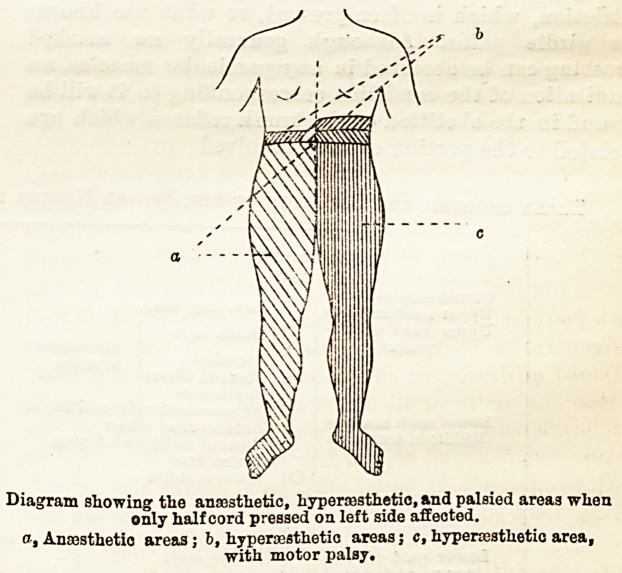


**Figure f2:**